# A Comparison between Transcriptome Sequencing and 16S Metagenomics for Detection of Bacterial Pathogens in Wildlife

**DOI:** 10.1371/journal.pntd.0003929

**Published:** 2015-08-18

**Authors:** Maria Razzauti, Maxime Galan, Maria Bernard, Sarah Maman, Christophe Klopp, Nathalie Charbonnel, Muriel Vayssier-Taussat, Marc Eloit, Jean-François Cosson

**Affiliations:** 1 INRA, UMR CBGP (INRA / IRD / Cirad / Montpellier SupAgro), Montpellier, France; 2 INRA, GABI, Domaine de Vilvert, Jouy-en-Josas, France; 3 INRA, Sigenae Group, GenPhySE, INRA Auzeville, Castanet Tolosan, France; 4 INRA, UMR Bipar, ENVA, ANSES, USC INRA, Maisons-Alfort, France; 5 PathoQuest SAS, Paris, France; 6 Ecole Nationale Vétérinaire d’Alfort, UMR 1161, Virologie ENVA, ANSES, INRA, Maisons-Alfort, France; 7 Pasteur Institute, Laboratory of Pathogen Discovery, Biology of Infection Unit, Inserm U1117, Paris, France; University of Tennessee, UNITED STATES

## Abstract

**Background:**

Rodents are major reservoirs of pathogens responsible for numerous zoonotic diseases in humans and livestock. Assessing their microbial diversity at both the individual and population level is crucial for monitoring endemic infections and revealing microbial association patterns within reservoirs. Recently, NGS approaches have been employed to characterize microbial communities of different ecosystems. Yet, their relative efficacy has not been assessed. Here, we compared two NGS approaches, RNA-Sequencing (RNA-Seq) and 16S-metagenomics, assessing their ability to survey neglected zoonotic bacteria in rodent populations.

**Methodology/Principal Findings:**

We first extracted nucleic acids from the spleens of 190 voles collected in France. RNA extracts were pooled, randomly retro-transcribed, then RNA-Seq was performed using HiSeq. Assembled bacterial sequences were assigned to the closest taxon registered in GenBank. DNA extracts were analyzed via a 16S-metagenomics approach using two sequencers: the 454 GS-FLX and the MiSeq. The V4 region of the gene coding for 16S rRNA was amplified for each sample using barcoded universal primers. Amplicons were multiplexed and processed on the distinct sequencers. The resulting datasets were de-multiplexed, and each read was processed through a pipeline to be taxonomically classified using the Ribosomal Database Project. Altogether, 45 pathogenic bacterial genera were detected. The bacteria identified by RNA-Seq were comparable to those detected by 16S-metagenomics approach processed with MiSeq (16S-MiSeq). In contrast, 21 of these pathogens went unnoticed when the 16S-metagenomics approach was processed via 454-pyrosequencing (16S-454). In addition, the 16S-metagenomics approaches revealed a high level of coinfection in bank voles.

**Conclusions/Significance:**

We concluded that RNA-Seq and 16S-MiSeq are equally sensitive in detecting bacteria. Although only the 16S-MiSeq method enabled identification of bacteria in each individual reservoir, with subsequent derivation of bacterial prevalence in host populations, and generation of intra-reservoir patterns of bacterial interactions. Lastly, the number of bacterial reads obtained with the 16S-MiSeq could be a good proxy for bacterial prevalence.

## Author Summary

The majority of human pathogens are of animal origin, i.e. zoonoses; both domestic and wild animals act as host reservoirs. Epidemiological surveys of wildlife may help to predict, prevent and control putative episodes of emerging zoonoses. Microbial diversity and their interactions at both the individual and population level may influence epidemiological infections. Developing generic approaches able to simultaneously detect multiple pathogens without any a priori information becomes essential. Here, we assess the relative efficacy of distinct next-generation sequencing (NGS) approaches to survey neglected zoonotic bacteria in rodent populations: RNA-sequencing (RNA-Seq) and 16S-metagenomics, with the latter resolved via two sequencing techniques, 454-pyrosequencing and MiSeq. The resulting data generated a thorough inventory of zoonotic bacteria in the rodent sample without any previous knowledge of their presence. We concluded that RNA-Seq and 16S-MiSeq are equally sensitive in bacterial genus detection. Nevertheless, only the 16S approach was able to determine bacterial diversity in each individual, which then permitted the derivation of bacterial prevalence and interaction patterns within host populations. We are persuaded that NGS techniques are very affordable candidates and could become routine approaches in future large-scale epidemiological studies.

## Introduction

A survey of infectious organisms revealed that 61% of human pathogens are of animal origin [1]. Generally, humans are accidental victims and dead-end hosts for zoonotic agents carried by both domestic and wild animal reservoirs. Rodents represent one of the major pathogen reservoirs responsible for a wide range of emerging zoonotic diseases in humans and livestock [2,3]. Rodent species are distributed across a vast range of habitats and often provide an interface between wildlife and urban communities, exposing humans and domestic animals to pathogens circulating in natural ecosystems. Surveys of rodents and their associated *pathobiome* [4] may help to predict, prevent and control putative episodes of emerging zoonoses. Thus, developing new approaches for pathogen detection without any prior knowledge of their presence is essential. This is vitally important, as numerous studies have emphasized the role of rodents in the transmission of both known and potential zoonotic agents, and also because the rodent microflora composition may influence the likelihood of transmitting infection [5,6]. Indeed, there is some evidence that interactions between pathogens can affect mammal infection risk [7]. Rodents infected by cowpox virus exhibit higher susceptibility to other microparasites such as *Anaplasma*, *Babesia* and *Bartonella* [8]. Conversely, infection with the hemoparasite *Babesia microtis*, reduces rodent susceptibility to *Bartonella spp*. [8] Multiple coinfections have also been described for Croatian rodents [9] hence a community-based ecological perspective is particularly relevant when studying zoonoses, both from epidemiological or evolutionary points of view [4]. Therefore it is crucial to assess microbial diversity in order to monitor endemic infections in natural populations, and also to reveal pathogen interactions within each reservoir.

Until now, the identification of pathogens in animal reservoirs has relied on individual case-by-case strategies, which are based on species-specific detection tests such as real-time quantitative PCR (qPCR), DNA arrays or antibody detection. All these approaches require a certain anticipation of the results, thus preventing the detection of microorganisms that are not known or sought after. Considering that we have a rather incomplete picture of microorganism diversity in reservoirs, it is highly likely that relevant pathogens may pass unnoticed. Thus the detailed description of entire pathogen communities is a fundamental necessity. However, this integrative scenario (i.e., complete screening of microbes in both hosts and vectors) has been impaired due to technological limitations. Nowadays the one-at-a-time approach is no longer feasible due to the high number of potential pathogens circulating in natural populations. Consequently there is a pressing need to develop generic approaches which are able to simultaneously detect and characterize large numbers of pathogens without any *a priori* information. Lately, next-generation sequencing (NGS) approaches combined with bioinformatics have revolutionized many fields of research including that of infectious diseases. We and others have demonstrated that NGS methods are highly efficient tools for detecting and characterizing new microorganisms in ticks [10,11], viruses [12], bacteria [13,14] and parasites [15]. Such sequencing methods differ primarily by the nature of the samples (RNA- or DNA-based), by the strategies to prepare the sequencing libraries and by the data analysis options used. There is a great number of NGS methods, and in this study we compare the main ones using RNA and DNA samples: transcriptomics and 16S metagenomics, respectively. Transcriptomics is based on the sequencing of the total RNA and provides a comprehensive view of a transcriptional profile at a given moment, thus reflecting the expression patterns of the pathogen community. 16S metagenomics is based on the sequencing of a DNA amplicon coding for the 16S rRNA gene common across all bacterial species, therefore allowing at once the amplification of all the bacterial species that infected the host. Such approaches offer great potential for large-scale epidemiological studies in wild animals, but as yet they have not been widely used in this context.

In this study, we evaluated the potential of NGS methods as tools for large-scale surveying of zoonotic pathogens carried by rodents. As stated earlier, certain pathogens can often remain undetected, either because they are as yet unknown, or simply because they are not expected in a particular reservoir species or geographic area. To address these issues, we combined several NGS approaches in order to establish a catalogue of zoonotic bacteria (without prior knowledge of their existence), which then allowed us to derive their prevalence in the host population. We also compared the efficiency of the two NGS approaches to detect zoonotic pathogens in epidemiological studies; the RNA-sequencing (RNA-Seq), and the 16S metagenomics processed with either 454 pyrosequencing or MiSeq technology.

## Methods

### Ethics statement

Animals were treated in accordance with the European Union legislation guidelines (Directive 86/609/EEC). The CBGP laboratory received approval (no. B 34-169-1) from the regional Head of Veterinary Services (Hérault, France), for rodent sampling, sacrifice, and tissue harvesting. Dr Cosson had authorization from the French Government to experiment on animals (no. C34-105).

### Sampling

The study area was located in the French Ardennes, a region endemic for many rodent-borne pathogens [16,13,17]. The sampling of bank voles (*Myodes glareolus*) was performed in autumn 2008 at ten trapping sites along an ~80 km transect line [18]. We used 190 bank voles for our analyses. None of the animals presented visible signs of diseases and the ratio of male/female and adult/young animals were merely equivalent in our sample set [18]. Once captured, animals were euthanatized by cervical dislocation, weighed, sexed and then dissected. In order to prevent cross contamination during dissection, we systematically alternated the use of several sets of dissecting instruments. After dissecting a rodent and also harvesting the distinct organs, the set used was soak in bleach for five minutes, rinsed with water and then in alcohol, while the next rodent was dissected with another set [19]. Organs were placed in RNAlater (Sigma, MO, USA) and immediately stored at -20°C for later analyses. In this study, we used exactly the same 190 bank voles to compare two different approaches: transcriptomics and 16S metagenomics, for detection of bacteria in rodents.

### Laboratory procedures

**Fig 1:**
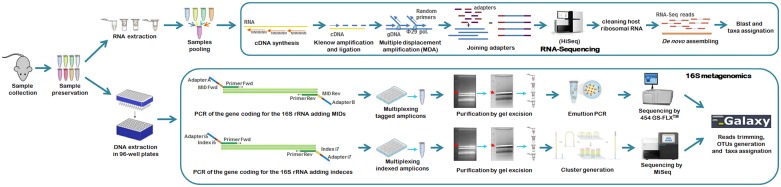
Flow chart of the NGS approaches used for bacteria detection. RNA sequencing processed with HiSeq (RNA-Seq) vs. 16S metagenomics processed with either 454-pyrosequencing (16S-454) or MiSeq (16S-MiSeq).

Total RNA was extracted from the spleen samples of 190 bank voles using the TRIzol/chloroform protocol as detailed by the manufacturers (Life Technologies, CA, USA). The integrity of RNA of the pool of samples was judged using an agarose gel. In addition the RNA integrity number (RIN) was assessed with Agilent’s 2100 Bioanalyzer (Agilent Technologies, Germany) software algorithm revealing an acceptable integrity of the RNA (RIN = 8.8). Genomic DNA was also extracted from the spleen of each bank vole using the 96-Well Plate Animal Genomic DNA Kit (BioBasic, ON, Canada) according to manufacturer’s instructions, with final elution into 100 μl water. To detect bacteria in these samples we used two NGS approaches: RNA-sequencing and 16S metagenomics. For the latter we analyzed DNA samples in parallel using two different NGS platforms, the 454 GS-FLX (Roche, Basel, Switzerland) and the MiSeq (Illumina, CA, USA). The main steps of both approaches are detailed below and in Fig 1.

High-throughput RNA-sequencing (RNA-Seq) was performed on an equimolar pool of all 190 RNA bank vole samples (Fig 1). Briefly, RNA was first retro-transcribed to cDNA, then randomly amplified by the bacteriophage φ29 DNA polymerase-based multiple displacement amplification (MDA) assay using random hexamer primers as described in [20]. Ligation and whole genome amplification (WGA) were performed with the QuantiTect whole transcriptome kit (Qiagen, Limburg, Netherlands) according to the manufacturer's instructions. The library was paired-end (2 x 101 bp) sequenced [20] with the HiSeq2000 (Illumina, CA, USA) obtaining 62 M of reads.

The 16S metagenomics approach was performed for each individual bank vole sample (190 in total). To obtain sequence data, two different NGS platforms were used: the *Roche* 454 GS-FLX pyrosequencing, or the *Illumina* MiSeq system (Fig 1). For 454-pyrosequencing, PCR amplification was performed on each rodent DNA sample using universal primers modified from Claesson *et al*. [21] (520-F: AYTGGGYDTAAAGVG; 802-R: TACCVGGGTATCTAATCC). These amplified the V4 hypervariable region of the bacterial 16S ribosomal RNA gene (16S rRNA), generating a 207 bp product, excluding primers. Amplicon lengths were designed to be comparable with MiSeq amplicons. Primers were tagged by adding 7 bp multiplex identifier sequences (MIDs) and 30 bp Titanium adapters to 5’ ends as described by Galan *et al*. [22]. Such adapters were required for emulsion PCR (emPCR) and subsequent 454 GS-FLX pyrosequencing using Lib-L Titanium Series reagents. We used the unique combination of 18 forward- and 16 reverse-primers containing distinct MIDs that permitted the amplification and individual tagging of 288 different 16S-amplicons. The tagged amplicons were then pooled, purified by AMPure XP beads (Beckman Coulter, CA, US), size selected by Pippin Prep electrophoresis (Sage Science, MA, USA), clonally amplified by emPCR and sequenced on a *Roche* 454 GS-FLX quarter picotiter plate. 454-pyrosequencing was subcontracted to Beckman Coulter Genomics (Danvers, MA, USA). For *Illumina* MiSeq sequencing, rodent DNA samples were amplified using universal primers modified from Kozich *et al*. [23] (16S-V4F: GTGCCAGCMGCCGCGGTAA; 16S-V4R: GGACTACHVGGGTWTCTAATCC), to amplify the bacterial 16S rRNA V4 hypervariable region, generating 251 bp products, excluding primers. These primers were dual-indexed by adding 8 bp-indices (i5 and i7) and *Nextera Illumina* adaptors (P5 and P7) as described by Kozich *et al*. [23]. We used a unique combination of 24 i5-indeces and 36 i7-indeces, this accredit the identification and hence the ability to multiplex 864 different amplicons. The pooled amplicon library was size-selected by excision following low-melting agarose gel electrophoresis and purified using the NucleoSpin Gel clean-up kit (Macherey-Nagel, PA, USA). DNA quantification was performed by quantitative PCR using the *KAPA* library quantification Kit (KAPA BioSystems, MA, USA) on the final library, prior to loading on the *Illumina* MiSeq flow-cell using a 500 cycle reagent cartridge and 2 x 251 bp paired-end sequencing.

#### Sequence analyses and taxonomic classification

RNA-Seq reads were trimmed according to their quality score. At the time of analysis, there was no published reference genome for *Myodes glareolus*, so vole sequences were removed from the analysis by subtracting sequences derived from *Rattus* and *Mus* databases using the SOAP2 aligner tool [24]. Then, *de novo* assembly was performed on all remaining reads (7.7 Mio), producing 112,014 bacterial contigs. Taxonomic assignment for contigs was achieved via successive sequence alignment using the non-redundant nucleotide and protein databases from NCBI and the BLAST algorithm. Contigs were assigned to the closest homolog taxon according to their identity percentage, and distant alignments were disregarded. Unambiguous assignments to specific taxons only occurred when percentage similarity between a contig (longer than 100 nt) and a specific taxon sequence was ≥ 95% (and lower when compared to other species). The 16S metagenomics data sets were processed using the Galaxy instance [25] (http://galaxy-workbench.toulouse.inra.fr/). To analyze the 16S-amplicon reads generated by 454 or MiSeq, two distinct pipelines were implemented using the Mothur program package [26], following the standard operating procedure of Patrick D. Schloss [27,23]. These pipelines were composed of several stages. The first corresponded to data pre-processing: for *Roche* 454-pyrosequencing, reads were de-multiplexed and primers discarded, for *Illumina* MiSeq, paired-reads were assembled. For both technologies, reads were then trimmed based on their length and quality score, and unique sequences were subsequently regrouped and chimeric sequences removed. To remove sequencing errors before sequences were associated with a taxonomic classification, pre-clustering at 3% dissimilarity threshold was performed. Taxonomic assignment was based on a naïve Bayesian classifier [28] using Bergey’s bacterial taxonomy [29] and the Ribosomal Database Project (RDP classifier) [30]. Arising from this procedure, 271,527 and 4,302,490 reads were assigned to bacteria using 454-pyrosequencing and MiSeq, respectively. As recommended by Claesson and his colleagues [31] we used a bootstrap cut-off value ≥ 60%, which allowed 94.5% of the reads to be correctly assigned to a bacterial genus when using the V4 region of 16S rRNA gene. Because the V4 hypervariable region has a higher degree of sequence conservation compared to other hypervariable regions, it has been speculated that this sequence may not be ideal for species differentiation [32], therefore, for such a reason, we analyzed our bacterial taxa at the genus level. Finally, we focused on those bacterial genera that included species known or suspected to be zoonotic. To this aim, we performed a systematic literature review [33,34,35,36,37] to identify zoonotic bacteria carried by rodents. Data deposited in the Dryad repository: http://dx.doi.org/10.5061/dryad.50125 [38].

#### Bacterial occurrence and prevalence

Taxon prevalence was calculated as the number of rodents positive for a particular bacterium, over the total number of rodents analyzed. Rodent samples were considered positive for a given bacterium when the number of reads exceeded five in that sample. We set the five-read threshold in order to minimize false positives due to potential taxonomic misidentification using the RDP classifier, and/or a possible read misassignment due to MIDs or indeces misidentification [31,39]. As this threshold value is quite arbitrary and deserves further investigation, we performed thorough validation tests. Accordingly, we repeated our analyses with two other threshold values, >1 read and >10 reads, and measured the impact of threshold value variation on results. Finally, rodent co-infection by several bacteria was assumed when more than five reads for each bacteria were recorded in the same rodent sample. For these calculations we used 16S-MiSeq data due to its higher coverage for each individual (mean = 23,440 reads/sample) compared to 454 data (mean = 1,454 reads/sample).

## Results

### Inventory of zoonotic bacterial genera

A total of 45 potential zoonotic bacterial genera were detected within the analyzed rodent samples (Table 1). We noticed remarkable congruence between RNA-Seq and 16S-MiSeq results, which detected 95.5% and 91% of 45 genera, respectively. Only a few genera were exclusively detected by either just one or the other approach, and had low read numbers (<90 reads for RNA-Seq and <545 reads for 16S-MiSeq), and a low prevalence of <4% positive rodents for 16S-MiSeq data (Table 1). In comparison, the 16S-454 approach was far less efficient, detecting only 53% of the 45 genera. Generally, zoonotic bacteria with prevalences less than 10% were not detected by the 16S-454. This is likely due to differences in sequencing depth for the various techniques, which resulted in 23,311 zoonotic bacterial reads using the Roche 454 GS-FLX (16S-454), 41,616 reads using the *Illumina* HiSeq (RNA-Seq), and 1,811,652 reads using the *Illumina* MiSeq (16S-MiSeq).

**Table 1:** Bacterial genera detected integrating zoonotic species. The number of bacterial reads obtained with each NGS approach are described, as well as some ecological information. RNA-sequencing processed with HiSeq (RNA-Seq) vs. the 16S metagenomics processed either with 454-pyrosequencing (16S-454) or with MiSeq (16S-MiSeq) are noted.

Bacterial genus		No. of reads			No. of samples with >5 16S-MiSeq reads
	Biology	RNA-Seq^◊^	16S-454	16S-MiSeq	
*Aeromonas*	Saprophytes living in humid environments, opportunistic animal pathogens	210	1	479	21
*Anaplasma*	Intracellular animal and human parasites, vectored by arthropods (ticks), responsible for mammal diseases	14	0	24	2
*Bacillus* *	Saprophytes in soil and water, some species are pathogenic for mammals (anthrax and food poisoning)	1,144	0	233	7
*Bartonella*	Intracellular parasite, vectored by arthropods (ticks, fleas, sans flies, mosquitoes), responsible for mammal diseases	275	21 898	1,725,562	166
*Bordetella*	Obligate parasites responsible for respiratory diseases in mammals (whooping cough)	994	0	20	0
*Borrelia*	Obligate animal parasites, vectored by arthropods (ticks, lice), responsible for Lyme disease and relapsing fever in mammals	373	45	566	4
*Brucella*	Intracellular parasites via direct transmission (food, aerosols), responsible for diseases in mammals	31	0	32	0
*Burkholderia* *	Saprophytes, some species are pathogenic for plants and animals	573	0	179	6
*Campylobacter*	Commensals in gut of many birds and mammals, opportunistic pathogens (food poisoning) for mammals	229	0	298	5
*Clostridium*	Common free-living bacteria of commercial interest as well as important pathogens (botulism, tetanus) for mammals	1,501	0	12	1
*Corynebacterium* *	Saprophytes of industrial use, some species are pathogenic (diphtheria, endocarditis) for mammals	467	21	5,025	82
*Coxiella*	Intracellular parasite of arthropods (endosymbionts of arthropods) and vertebrates, transmitted by aerosols, mucus and rarely by ticks, agent of Q fever	21	0	0	0
*Ehrlichia/Neoehrlichia*	Intracellular parasites of vertebrates, vectored by arthropods (ticks), responsible for diseases in mammals	40	0	17	1
*Enterococcus*	Commensals of digestive tract, opportunistic pathogens (septicemia, urinary tract infection) of mammals	228	0	162	3
*Eubacterium*	Commensals in vertebrates gut, opportunistic pathogens in humans	221	0	40	12
*Francisella*	Intracellular parasite of arthropods and vertebrates, transmitted by direct contact and vectors (ticks, mosquitoes, flies), responsible for mammal diseases (tularemia)	71	0	0	0
*Granulicatella*	Commensals of mucosal surfaces, opportunistic pathogens	0	0	545	8
*Haemophilus*	Commensals of mucosal surfaces, opportunistic pathogens	76	25	1,121	17
*Helicobacter*	Pathogens living in stomach and liver, responsible for mammal diseases (chronic gastritis, cancer)	25,944	35	6,532	81
*Klebsiella*	Saprophytes in soil and water, commensals of gastrointestinal tract, opportunistic pathogen responsible for septicemia, pneumonia in mammals	110	1	30	0
*Legionella* *	Saprophytes in soil and water, some species are agents of mammal diseases (pneumonia)	68	54	5,065	105
*Leptospira*	Saprophytes in humid environments, many species are agents of mammal diseases (leptospirosis)	330	183	1,936	4
*Listeria*	Saprophytes in soil and water, opportunistic pathogen causing serious diseases in mammals (listeriosis)	125	1	154	6
*Mannheimia*	Saprophytes of the upper respiratory tract, opportunistic pathogens in mammals (pneumonia)	6	2	266	4
*Micrococcus* *	Saprophytes in soil and water, commensals of skin, opportunistic pathogens	26	8	1,231	39
*Moraxella*	Commensals of mucosal surfaces, opportunistic pathogens (lower respiratory tract infections)	8	211	106	1
*Mycobacterium*	Saprophytes in humid environments, includes pathogens known to cause serious diseases in mammals (tuberculosis, leprosy)	1,195	0	735	20
*Mycoplasma*	Saprophytes, commensals of mucosal surfaces and parasites responsible for mammalian diseases (pneumonia, arthritis, cancer)	1,260	0	210	6
*Neisseria*	Commensals of mucosal surfaces, some species are pathogenic (gonorrhea, septicemia) for mammals	3,399	7	1,183	15
*Neochlamydia*	Endosymbiont of the amoebae, causative agent of diseases in mammals.	0	1	65	2
*Nocardia*	Saprophytes in soil, commensals of oral cavity, opportunistic pathogens	95	6	30	1
*Orientia*	Intracellular animal parasites, transmitted by vectors (trombiculid mites), responsible for human disease (scrub typhus)	16	95	7,806	22
*Pasteurella*	Commensals of mucosal surfaces, many species are opportunistic mammalian pathogens, infection acquired from animal bites and direct contact	40	192	69	2
*Rhodococcus* *	Saprophytes in soil and water, one species is pathogenic for animals (pneumonia)	321	3	8,977	147
*Rickettsia*	Intracellular parasite, transmitted by vectors (ticks, fleas, chiggers, lice), responsible for human diseases (spotted fever, typhus)	157	1	770	5
*Salmonella*	Saprophytes in humid environments, opportunistic pathogen (typhoid fever, food poisoning)	90	0	0	0
*Shigella/Escherichia*	Commensals of the digestive and urinary tracts, opportunistic pathogens (diarrhea to dysentery)	70	1	676	12
*Spiroplasma*	Symbionts in the gut or insect hemolymph, few species are pathogenic for mice (cataracts and neurological damage)	67	2	20,449	18
*Staphylococcus* *	Saprophytes in soil, commensals of skin and mucosal surfaces, opportunistic pathogens (septicemia, food poisoning)	235	0	6,429	95
*Stenotrophomonas* *	Saprophytes in soil and opportunistic pathogens of respiratory or urinary tracts	84	0	3,463	72
*Streptococcus* *	Saprophytes in soil and water, commensals of skin and mucosal surfaces, opportunistic pathogens (septicemia, meningitis, pneumonia)	687	34	5,102	69
*Treponema*	Commensals of mucosal surfaces, some species responsible for syphilis and skin infections	190	9	382	7
*Ureaplasma*	Commensals of urogenital tracts, opportunistic pathogens	139	0	27	1
*Vibrio*	Saprophytes in humid environments, opportunistic pathogens (food-borne infection)	420	0	25	1
*Yersinia*	Saprophytes in soil and water, some species are pathogenic for mammals (plague, yersiniosis)	64	0	5,616	120
Total		41,614	22,836	1,811,649	190

* These bacterial genera have been identified as a contaminant of DNA extraction kit reagents and ultrapure water systems, which may lead to their erroneous appearance in microbiota or metagenomic datasets [43](Salter *et al*. 2014)

Most well-known pathogens for which European rodents are reservoirs were detected, notably *Bartonella*, *Rickettsia*, *Borrelia*, *Neoehrlichia* and *Anaplasma*. Whilst *Francisella* and *Coxiella* were only found using RNA-Seq, with low numbers of recorded reads. Nevertheless, we also detected the genus *Orientia*, for which the only known species (*O*. *tsutsugamushi*) is a rodent-borne bacterium responsible for scrub typhus in Asia [40]. Non-arthropod-borne bacterial genera were also detected, including pathogens responsible for zoonotic diseases in humans. High numbers of *Leptospira* were recorded by both RNA and DNA approaches. *Helicobacter*, *Spiroplasma*, *Haemophilus*, *Mycobacterium* and *Neisseria* were also reported with high numbers of reads. A large number of bacterial commensals and saprophytes that could become opportunistic pathogens under certain conditions, were also detected, including *Aeromonas*, *Bordetella*, *Brucella*, *Campylobacter*, *Clostridium*, *Enterococcus*, *Eubacterium*, *Granulicatella*, *Klebsiella*, *Listeria*, *Mannheimia*, *Moraxella*, *Mycoplasma*, *Nocardia*, *Pasteurella*, *Shigella*, *Treponema*, *Ureaplasma* and *Vibrio*. Furthermore, we also detected a number of opportunistic pathogens with very high numbers of reads and in a large number of rodents (Table 1). Bacteria which frequently contaminate laboratory reagents, namely *Corynebacterium*, *Legionella*, *Micrococcus*, *Rhodococcus*, *Staphyloccocus*, *Stenotrophomonas* and *Streptococcus*, were notably abundant in our samples. Accordingly, we identified reads from those bacteria in our 16S-MiSeq negative controls, most notably *Corynebacterium* (4% of the reads obtained for this bacterium were identified in the negative controls), *Legionella* (0.3%), *Rhodococcus* (2.2%) and *Staphyloccocus* (4%).

### Identification to a bacterial species level

In some cases, RNA-Seq data resulted in the identification of bacteria to the species level, which was not feasible for 16S metagenomics data with poorer accuracy at this taxonomic level. Species assignment using RNA-Seq data occurred for 7 distinct genera, leading to 11 bacterial species: *Bartonella birtlesii* (with a 100% nucleotide identity), *B*. *vinsonii* (100% identity), *B*. *doshiae* (98% identity), *Helicobacter pylori* (99.9% identity), *Burkholderia cepacia* (99% identity), *Bacillus thuringiensis* (97.2% identity), *Eubacterium siraeum* (97.2% identity), *Klebsiella pneumonia* (97.6% identity), *Mycoplasma haemomuris* (98.2% identity), *M*. *haemocanis* (96.5% identity) and *M*. *haemofelis* (96.6% identity).

### Relative abundance of zoonotic bacteria

The number of bacterial reads varied greatly according to the bacterial genera considered and the NGS approach used (Table 1 and S1 Fig). In particular, 16S metagenomics generated a large majority of *Bartonella* reads. They represented 94% of zoonotic bacterial reads produced using 16S-454, 95% using 16S-MiSeq, while only 0.7% via RNA-Seq; which, respectively, equated to 8.1%, 40.1%, and 0.2% of total bacterial reads (or 0.8% after applying genome length corrections described by Mortazavi and co-workers [41]). It should be kept in mind that RNA-Seq generates reads from random amplifications of a fragmented library, which produces length bias as longer genomes are more regularly amplified, and thus present higher counts in contrast to shorter genomes [41]. Hence RNA-Seq can only be informative about relative transcript abundance, unless additional data, such as “spike-in” transcript levels, are added for absolute quantification. Overall, the relative abundance of zoonotic bacteria genera was more evenly balanced with RNA-Seq data than that obtained using 16S metagenomics data (S1 Fig). Accordingly, we found no significant correlation between the numbers of bacterial reads produced by RNA-Seq or 16S metagenomics (RNA-Seq *vs*. 16S-454: R^2^ = 0.019, *P* = 0.688; RNA-Seq *vs*. 16S-MiSeq: R^2^ = 0.015, *P* = 0.206; S2 Fig).

### Prevalence of bacterial DNA-positive animals

To estimate the bacterial prevalence within our sample, we reported the number of positive rodents (at least five 16S-Miseq reads) for each of the 45 zoonotic bacterial genera detected. We found a large variation in prevalence across bacterial genera. Among vector-borne bacteria, *Bartonella* was the most prevalent (>5 reads in 89% of the rodents) followed by *Orientia* (12%), *Borrelia* (4%), *Rickettsia* (3%), *Neoehrlichia* (1%) and *Anaplasma* (1%). Among other bacteria, *Helicobacter* was detected in 48% of rodents, *Mycobacterium* in 15%, *Neisseria* in 14%, *Haemophilus* in 13%, *Spiroplasma* in 11%, *Mycoplasma* in 5%, and *Leptospira* in 2%. Furthermore, the presence of bacteria known to contaminate laboratory reagents was notably high, including *Rhodococcus* (82%), *Legionella* (63%), *Staphylococcus* (58%), *Corynebacterium* (49%), *Streptococcus* (45%), *Stenotrophomonas* (42%) and *Micrococcus* (29%), thus likely suggesting that these bacteria were of contaminant origin rather than actually infecting rodents.

**Fig 2:**
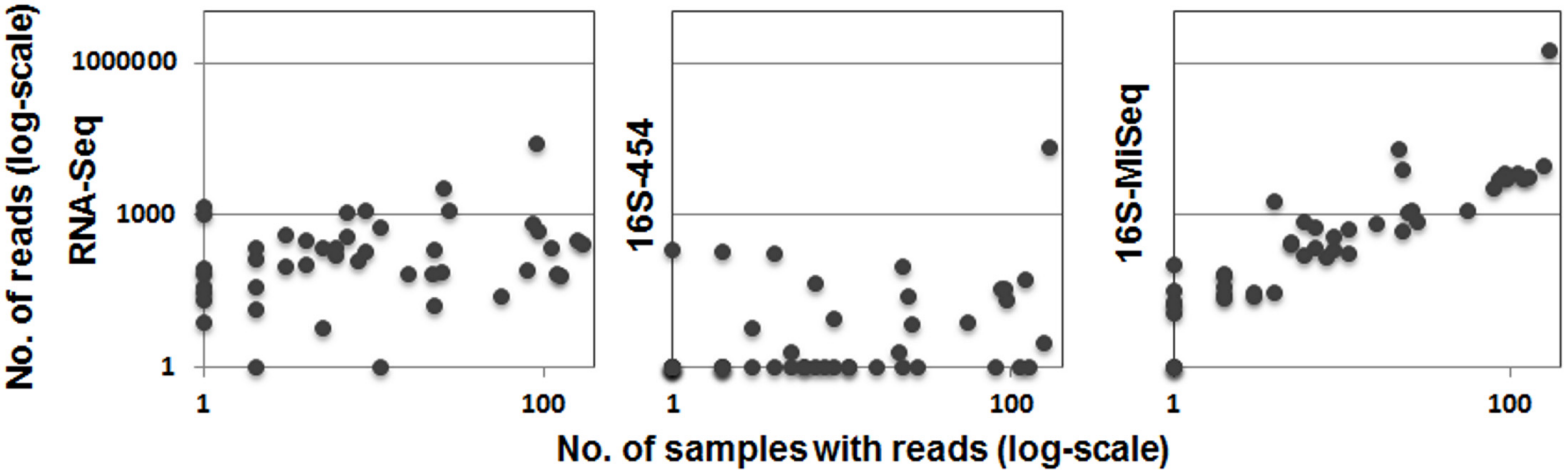
Correlation between the number of bacterial reads versus the number of rodent samples with at least five reads. Number of reads are those from RNA sequencing processed with HiSeq (RNA-Seq), and 16S metagenomics processed with either 454-pyrosequencing (16S-454) or MiSeq (16S-MiSeq). The number of positive samples is taken from 16S-Miseq results. Correlation coefficients (R^2^) and statistical significance (P) are 0.069 (P = 0.069), 0.088 (P = 0.027) and 0.763 (P<0.001), respectively.

Correlations between zoonotic bacterial read number and their prevalence were weak using both RNA-Seq (R^2^ = 0.053, *P* = 0.069; Fig 2) and 16S-454 approaches (R^2^ = 0.088, *P* = 0.027; Fig 2), whilst the correlation was positive and highly significant for the 16S-MiSeq methodology (R^2^ = 0.763, *P*<0.001; Fig 2). The use of different threshold values to validate positivity (>1 read, >5 reads and >10 reads) did not influence bacterial genera detection across the whole sample. However, it did change results at an individual level, thus directly affecting prevalence estimates. We observed an average 2% increase in prevalence rates when the threshold was lowered to one read, and an average 4% decrease when fixed to 10. Note that these threshold values did not affect the relationships observed between the number of bacterial reads and their prevalence (Threshold >1: RNA-Seq: R^2^ = 0.035, *P* = 0.113; 16S-454: R^2^ = 0.067, *P* = 0.047; 16S-MiSeq: R^2^ = 0.773, *P*<0.001. Threshold >10: RNA-Seq: R^2^ = 0.040, *P* = 0.099; 16S-454: R^2^ = 0.094, *P* = 0.023; 16S-MiSeq: R^2^ = 0.747, *P*<0.001; S3 and S4 Figs).

### Co-infection

**Fig 3:**
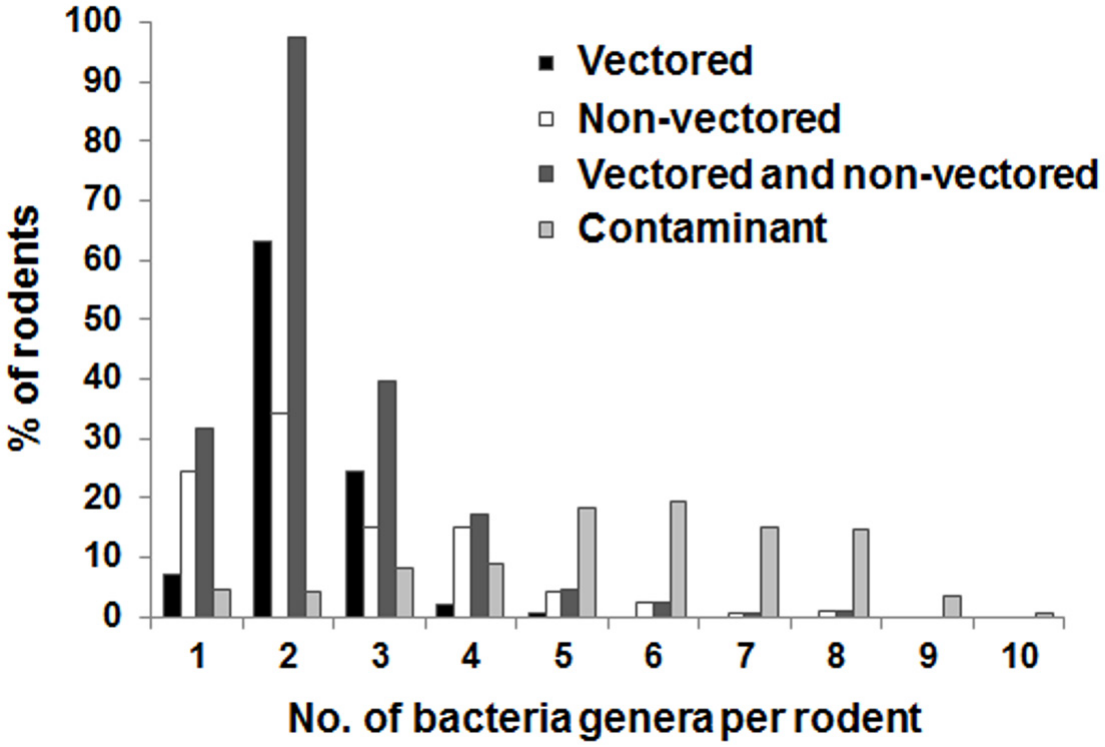
Distribution of the number of bacteria genera per rodent according to their transmission pathway (vectored vs. non-vectored bacteria). Contaminants of laboratory reagents are also shown. The results shown are from the MiSeq data. Prevalence is estimated using the number of rodent samples with at least five reads.

Since the 16S-MiSeq approach has highly efficient bacterial detection with the option of multiplexing, its results proved suitable for calculating bacterial prevalence but also deriving coinfections. Bacterial genera suspected to be contaminants (see above in the text and Table 1) were analyzed independently. We also separately analyzed vectored bacteria (i.e. transmitted via arthropods) and non-vectored bacteria because of their very different transmission routes and epidemiology. The co-infection rate for both vectored and non-vectored bacteria was 27% and 39% respectively (Fig 3). The mean number of bacteria per rodent was comparable for bacteria either transmitted via the environment (mean = 1.5 bacteria genus/rodent) or by arthropods (mean = 1.4 bacteria/rodent). The mean number of contaminant bacteria per rodent was high (mean = 4.4). However, the two other tested rodent positivity threshold values for each given bacterium did not strongly affect these results. An average increase of 0.6 bacteria per rodent was observed for the one read threshold, compared to an average decrease of 0.3 bacteria per rodent for the 10 read threshold (S5 and S6 Figs).

## Discussion

Recently a number of studies have used random DNA-based [42], RNA-based [13] or 16S-based NGS strategies [14] to generate global pictures of wildlife-borne bacteria. However up until now, the pros and cons of these strategies have not been directly compared. Here we performed whole transcriptome (RNA-sequencing) and 16S metagenomics analyses on the same sample set of 190 bank voles. Below we discuss the advantages or drawbacks associated with each approach, as well as comparing their efficacy for generating bacterial inventories (Table 2). We also evaluated their usefulness in deriving bacterial prevalence within rodent populations, as well as co-infection rates within individual rodents.

**Table 2:** Pros and cons of different NGS approaches used for epidemiological surveying of bacterial pathogens. RNA-sequencing processed with HiSeq (RNA-Seq) vs. 16S metagenomics processed with either 454-pyrosequencing (16S-454) or MiSeq (16S-MiSeq).

	RNA-Seq	16S metagenomics	
	Hiseq 2000	MiSeq	454
Coverage:			
Catalog of bacterial genera	High	High	Poor
Completeness of catalog	91%	89%	41%
Completeness of databases	Moderate	High	High
Taxonomic accuracy	Species	Genus	Genus
Resolution of sample sequencing^1^	Pool	Individual	Individual
Multiplex capability	Low	High	Moderate
Prevalence estimates^2^	Poor	Accurate	Poor
Bacterial interaction studies^2^	None	Allowed	Poor
Ratio reads from bacteria/reads from host^3^	Low	High	High
Laboratory costs			
Price / lane	≈ 5 000 €	≈ 1 200 €	≈ 3 400 €^4^
Price / Mb	≈ 0.008 €	≈ 0.2 €	≈ 5 €
Sequencing characteristics			
Output data / lane	40 Gb	6 Gb	0.1 Gb^4^
Reads / lane	200–300 M	12 M	0.2 M^4^
Homopolymer errors	Low	Low	High
Read length	2x101 bp	2x251 bp	≈ 400 bp
Time/run	14 days	39 hours	10 hours

^1^ While theoretically possible, getting data for individual samples seems unaffordable for cost reasons;

^2^ Depends on the ability to multiplex large numbers of samples with concomitant high sample read numbers;

^3^ RNA-Seq produces large numbers of non-bacterial sequences (i.e. host, parasites and viruses);

^4^ Price and throughput for one region of a 4 region gasket PicoTiterPlate 454 Titanium run (PTP)

### Inventory of bacteria identified in rodents

We found that the bacterial genera detected by both RNA-Seq and 16S-MiSeq were remarkably congruent. Contrastingly, the 16S-454 was far less efficient as zoonotic bacteria with low prevalences were not detected. This is very likely due to differences in sequencing depth for each of the techniques used.

Most of the bacterial genera detected in the rodent samples were expected, i.e. already known to be hosted by rodents within the geographic area (Les Ardennes region, NE France). The high number of *Leptospira* RNA and DNA reads confirmed the important role of wild rodents in the circulation of leptospires in natural habitats. Likewise the high number of *Helicobacter*, *Spiroplasma*, *Haemophilus*, *Mycobacterium*, and *Neisseria* reads suggested considerably high infection rates for such bacteria in wild rodents. The high abundance of *Yersinia* reads could also indicate high and regular infection by *Yersinia pseudotuberculosis*, a well-known rodent parasite, yet *Yersinia* species are also common saprophytes of soils and water and their presence in our samples could also result from contamination. This point deserves to be further studied. The detection of bacterial commensals and saprophytes from RNA extracts suggests that these microorganisms were actively replicating in rodent spleens, therefore indicating effective infection of rodents by these bacteria in natural habitats. *Corynebacterium*, *Legionella*, *Micrococcus*, *Rhodococcus*, *Staphyloccocus*, *Stenotrophomonas* and *Streptococcus* were abundant, yet their actual presence in rodent spleens remains dubious as these genera are known to be frequent contaminants of nucleic acid extraction reagents and ultrapure water systems [43].

The use of these NGS approaches allowed us to highlight unforeseen bacteria in our rodent sample, either because the bacterium was not previously observed in the studied geographic area or because it was not expected in wild rodents. This was the case for *Orientia*, *Helicobacter*, and *Spiroplasma*.

#### Orientia

the causative agent of scrub typhus, for which the only known species *O*. *tsutsugamushi* is a rodent-borne bacterium responsible for Asian scrub typhus [40]. It is transmitted to humans by the bite of infected chigger mites (primarily *Leptotrombidium spp*.) [44]. In Asia, approximately one million cases of scrub typhus occur annually, where it is probably one of the most underdiagnosed and underreported febrile illnesses requiring hospitalization [45], with an estimated 10% fatality rate unless treated appropriately. Formerly thought to be geographically restricted to Asia, the *Orientia* bacterium has never before been reported in Europe. Phylogenetic analyses of the V4 sequences generated by the MySeq experiment suggest that the bacteria detected in our European voles are quite divergent from *Orientia tsutsugamushi*, and could represent a new species or lineage [46]. This example highlights the potential of new NGS tools for the surveillance of neglected diseases in localities where they do not appear on the public health service radar.

#### Helicobacter

With the exception of *Helicobacter pylori* which has been intensively studied [47], other *Helicobacter* species are neglected in animal and human epidemiological studies. However, non-*pylori Helicobacter* species (NPHS), which are naturally found in mammals and birds, have been detected in human clinical specimens, thus the role of NPHS in veterinary and human medicine is becoming increasingly recognized [36,48,49]. Concerning rodents, researchers have isolated at least eleven NPHS species liable to cause health disorders in domestic rodents like mice, rats, and hamsters (*H*. *hepaticus*, *H*. *muridarum*, *H*. *bilis*, *H*. *rodentium*, *H*. *typhlonius*, *H*. *ganmani*, *H*. *trogontum*, *H*. *cinaedi*, *H*. *cholecystus*, *H*. *aurati*, and *H*. *mesocricetorum*).

#### Spiroplasma

This diverse genus is associated with many host plants and arthropods, particularly insects. Many studies have shown that *Spiroplasma*-arthropod associations are common [50], and this genus has occasionally been reported as pathogenic for mice and cattle [51]. Up to now, there have been no reported cases of *Spiroplasma* presence in natural rodent populations.

### Bacterial contaminants of laboratory reagents

Recent work by Salter and his colleagues [43] highlighted the confounding effect on metagenomic studies of bacterial contamination from DNA extraction kits and other laboratory reagents. Contaminating DNA was demonstrated to be ubiquitous in commonly used DNA extraction kits, and to vary greatly in composition between different kits and kit batches. This contamination could critically impact the results of many metagenomic studies. Moreover Salter *et al*. [43] stressed that this impact would potentially be more severe when working on samples containing low microbial biomass and/or low total DNA. This could be the case for our biological samples because high bacterial loads are not expected in rodent spleens, unless the animals were heavily infected. In accordance with Salter *et al*. [43] we had indirect evidence for contamination of our samples by potentially pathogenic bacterial species like *Staphylococcus* and *Streptococcus*. The detection of contaminating bacteria with both RNA-Seq and 16S metagenomics proves that such bacteria are actively replicating, although their presence could result from both contamination of our samples by laboratory reagents and/or true rodent infections (at least for some of them). Distinguishing between those two possibilities seems difficult, if not impossible. In any case, our results urge epidemiologists to be cautious when deducing animal infection by the above bacterial species when using DNA-based approaches. We suggest that blank controls should be systematically introduced at different experimental stages throughout metagenomics studies. This becomes especially relevant for epidemiological studies where some important potential pathogenic bacterial genera are also common contaminants of laboratory reagents.

### Number of reads and relative bacterial abundance

We observed a lack of correlation between the numbers of bacterial reads produced by the different NGS approaches, suggesting that this parameter is a poor predictor of relative bacterial abundance. This major difference in read number arising from the various approaches could be due to several reasons, as discussed below:

Sequencing depth (the average number of times each base in the genome is sequenced) and sequencing coverage (the percentage of the genome that is covered by sequenced reads) varied among the three NGS techniques: the *Roche* 454 GS-FLX and the *Illumina* MiSeq and HiSeq; the latter being the most powerful in terms of amount of data generated i.e. both sequence depth and coverage. In this case HiSeq was used to perform whole transcriptome sequencing (RNA-Seq) of an RNA sample pool extracted from 190 rodent spleens, for this reason only a portion of the large number of obtained reads identified bacteria (reads corresponding to viruses, protozoa, and rodents were not analyzed in this study). In contrast, the alternative 16S metagenomics approach, (performed using both 454-FLX and MiSeq), was used to specifically amplify bacterial sequences. Thus we analyzed the totality of the reads obtained. In this way we obtained 271,257 bacterial reads using the *Roche* 454 GS-FLX (16S-454), 112,014 reads using the *Illumina* HiSeq (RNA-Seq), and 4,302,490 bacterial reads using the *Illumina* MiSeq (16S-MiSeq).

The process of genome amplification might also explain the differences observed with regard to the number of reads obtained. The approaches compared here used different template amplification strategies, and their performance could impact the number of reads generated. The *Roche* technology utilized emulsion PCR, whilst *Illumina* technology employed clonal bridge amplification. In addition, RNA-Seq used random primers permitting the amplification of any kind of DNA sequence, whilst the 16S approach is based on universal primers that likely unevenly target different bacterial species/genera. In this study, the *Roche* and *Illumina* 16S metagenomics analyses targeted the same 16S rRNA hypervariable region, but different universal primers were used depending on the sequencing technology (*Roche* or *Illumina*) and indeed the performance of either primer set may influence the amplification of certain bacteria species/genera. Therefore, the choice of these universal primers is crucial for the performance of such studies [52].

Variation in 16S genomic copy number among bacterial organisms may affect the relative abundance of the different bacteria using the 16S approach. 16S rRNA copy number varies greatly between species, ranging from 1 to 15 [53]. Consequently, variation in relative 16S gene abundance within a rodent sample can either reflect variation in the abundance of different bacterial organisms, or variation in 16S gene copy number among those organisms. This factor is of special importance when 16S metagenomics data is used to quantify taxa.

Additionally, specific biological processes of each bacterial species could also play a role in the presence and subsequent amplification and detection of such bacterial organisms in rodent spleens. For example, we were surprised by the huge difference in the relative abundance of *Bartonella* reads provided by the 16S-MiSeq (95%) vs. RNA-Seq (<1%). The most likely hypothesis is related with (what is known about) the biology of *Bartonella* within its mammalian host [54]. The currently accepted model holds that immediately after infection, *Bartonella* colonizes an unknown primary niche of mammalian host, most likely vascular endothelial cells. Every five days, some of the bacteria in the endothelial cells are released into the blood stream, where they infect erythrocytes. Then bacteria invade a phagosomal membrane inside the erythrocytes, where they multiply until they reach a critical population density. At this point, they simply wait until they are taken up with the erythrocytes by a blood-sucking arthropod. The spleen plays important roles with regard to erythrocytes. It removes old erythrocytes and holds a reserve of erythrocytes that are highly infected by non-replicating *Bartonella*, which do not produce RNA molecules. Moreover, due to its central role in recycling erythrocytes, the spleen could also store a large amount of degraded DNA of dead *Bartonella*. The cumulative effect of both processes might presumably explain the huge difference in relative abundance of *Bartonella* reads detected by 16S-MiSeq vs. RNA-Seq. The choice of organ to be studied likely has an important impact on the detection or misdetection of a given bacteria, and subsequently on our understanding of the composition of bacterial communities within hosts.

Finally, databases used for taxonomic classification may also be of significant importance when establishing bacterial inventories. The resulting taxa classification depends on available reference sequences and the taxonomic hierarchy used. Taxonomic assignation of RNA-Seq data was achieved via the BLAST algorithm against the NCBI database. Homology of ≥ 95% to an archived taxon permitted the classification of contigs. Consequently, divergent contigs were not taxonomically assigned; nevertheless this approach was able to classify more bacteria than with the 16S approach. For 16S data we used the RDP classifier, as the hypervariable region of our choice (V4) was better represented in that database than in other ribosomal databases [55]. It is likely that using other databases, i.e. Silva [56] or GreenGenes [57], would uncover other taxa that are as yet undetected by the RDP classifier. Hence, Werner and his colleagues [58] evaluated the impact of major ribosomal databases on bacterial taxonomic assignation. We did the same and discovered that *Mycoplasma*, which was detected at low levels using the RDP classifier, was copiously recorded (228,081 reads) when using the Silva database.

### Accuracy of taxonomic assignation

An important limitation of the approaches performed here is the accuracy level of the taxonomic assignation; to some extent, RNA-Seq allows taxa classification at a species level whilst 16S metagenomics classification is generally restricted to the genus level. For 16S metagenomics data, taxonomic assignation accuracy is limited by the barcode chosen to discriminate bacterial organisms. The 16S rRNA gene is approximately 1550 base pairs long, and difficult to sequence in its totality using current high-throughput sequencing methods. Although assembly steps do exist [59], they are not frequently used because they increase experimental complexity and cost. Instead, a portion of the 16S rRNA gene is usually amplified using specific sets of universal primers. The nine hypervariable (V) regions of the 16S rRNA gene differ between species, and depending on the V region chosen, one can discriminate some species but not others. Hence the use of different V regions influences operational taxonomic unit (OUT) clustering, suggesting caution when analyzing these data [27]. For this study we used the V4 hypervariable region which has poor resolution below the genus level [31] but a sequence length compatible with current sequencing technologies. Alternately, RNA-Seq has a higher potential for providing accurate bacterial-species assignation as recently shown by Vayssier-Taussat and colleagues [13], although it is currently limited by the lack of comprehensive genomic databases. Up to now only a small fraction of identified bacteria have been sequenced in their entirety, but owing to the fact that more bacteria are sequenced each year, this limitation should be mitigated in the future, facilitating more accurate bacterial taxonomic assignation.

In conclusion, the NGS methodologies presented here should be seen as effective means by which initial screening of bacterial communities can be performed in very large biological samples, either in populations (RNA-Seq) or individually (16S metagenomics). Based on these preliminary results, other methods could then be employed for bacterial species-level assignment. This may involve the use of PCR assays with bacterial genus-specific primers followed by amplicon sequencing as commonly used for *Bartonella* [60] or *Rickettsia* [61] species identification, or the use of qPCR assays based on bacterial species-specific primers [62]. In contrast to these specific approaches, NGS techniques have the outstanding advantage of being non-specific, thereby allowing the description of unexpected or potentially novel bacteria. Instead of being considered as alternatives, these approaches should be thought of as complementary.

### Bacterial prevalence estimates

It is tempting to derive bacterial prevalence using 16S-MiSeq data, since RNA-Seq does not provide individual sample information and 454-pyrosequencing is much less effective. The vector-borne bacterial prevalences estimated in this study are comparable to those observed in previous studies of wild rodents. *Bartonella* was the most prevalent in the rodent population [54] but other less predominant bacteria were also detected circulating in the population, such as *Borrelia* [63], *Rickettsia* [64], *Neoehrlichia* [65] and *Anaplasma* [66]. We however perceived that this strategy requires improved documentation. Defining an appropriate infection positivity threshold for individuals seems crucial, although we observed that this has only a slight impact on the results when using different threshold values. Choosing a correct threshold should rely on thorough analyses of potential biases, in particular those caused by incorrect sample read assignments and taxonomic misidentification. Such evaluation requires the performance of complementary experiments. Likewise the comparison of the 16S-MiSeq approach with PCR and qPCR-based approaches for specific bacteria needs to be documented to give a comprehensive picture of the pros and cons of those approaches for epidemiological surveys in terms of sensitivity and specificity.

Utilizing 16S-MiSeq read number as a reliable predictor of bacterial prevalence opens exciting perspectives for large-scale epidemiology. For instance, the monitoring of bacterial zoonotic agents in space and time over large geographic areas could be implemented via the analysis of population pools rather than per individual vectors and/or reservoirs. Such a strategy, which still needs to be thoroughly evaluated, would dramatically increase the number of monitored locations for the same amount of field and laboratory effort.

### Perspective: A general strategy for epidemiological survey

The results obtained by these NGS approaches allowed us to generate an almost complete inventory of potentially zoonotic known bacteria in rodent samples without any *a priori* on their presence. In addition, the use of multiplexing techniques granted us the ability to screen these microorganisms in each individual rodent, while the experimental costs remained compatible with cohort studies. However, one important limitation is the low accuracy of species-specific taxonomic determination. When this constraint is managed, NGS methods could be utilized for pre-screening, prior to species-specific tests using classical PCR and/or qPCR approaches. We are convinced that following their recent development, NGS techniques are ideally suited for routine implementation in future large-scale epidemiological studies. Their application should not be restricted to rodents, and wider study designs based on the sampling of reservoir and vector communities within specific areas would give important information about epidemiological cycles for poorly known bacteria. Complementarily, we showed that NGS can provide suitable datasets for the study of microorganism interactions. To predict and control the etiological agents of diseases in natural populations it is essential not only to understand host-parasite interactions but also the entire interactions of microorganism communities. We believe that the use of NGS techniques will pave the way for greater understanding of this field.

## Supporting Information

S1 Fig Relative proportion of the number of reads for each bacterial genus with different NGS approaches. RNA sequencing processed with HiSeq (RNA-Seq) vs. 16S metagenomics processed with either 454-pyrosequencing (16S-454) or MiSeq (16S-MiSeq).
(TIF)

S2 Fig Correlation between the number of bacterial reads produced by the different NGS approaches. RNA sequencing processed with HiSeq (RNA-Seq) vs. 16S metagenomics processed with either 454-pyrosequencing (16S-454) or MiSeq (16S-MiSeq). Correlation coefficients (R2) and statistical significance (P) are 0.019 (P = 0.688), 0.015 (P = 0.206) and 0.293 (P<0.001), respectively.
(TIF)

S3 Fig Correlation between the number of bacterial reads versus the number of rodent samples with at least one read. (TIF)

S4 Fig Correlation between the number of bacterial reads versus the number of rodent samples with at least ten reads. (TIF)

S5 Fig Distribution of the number of bacteria genera per rodent according to their transmission pathway (vectored vs. non-vectored bacteria). Contaminants of laboratory reagents are also shown. The results shown are from the MiSeq data. Prevalence is estimated using the number of rodent samples with at least one read.
(PDF)

S6 Fig Distribution of the number of bacteria genera per rodent according to their transmission pathway (vectored vs. non-vectored bacteria). Contaminants of laboratory reagents are also shown. The results shown are from the MiSeq data. Prevalence is estimated using the number of rodent samples with at least ten reads.
(PDF)
