# Supplementary figures and images for: A Comparison between Transcriptome Sequencing and 16S Metagenomics for Detection of Bacterial Pathogens in Wildlife
Source: PLoS Negl Trop Dis. 2015 Aug 18;9(8):e0003929. doi: 10.1371/journal.pntd.0003929 (PMC4540314; doi:10.1371/journal.pntd.0003929)

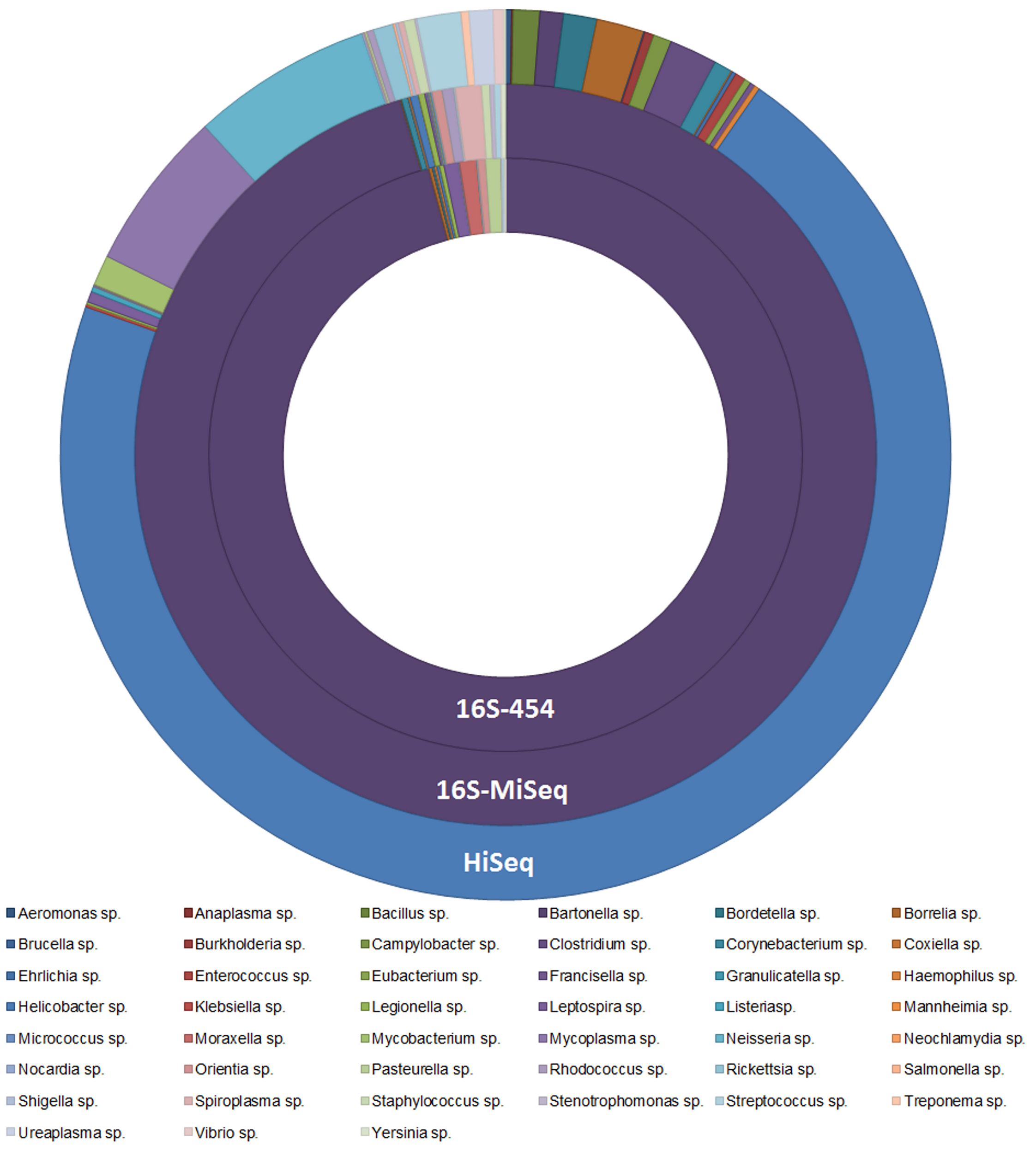

Supplement: S1 Fig — RNA sequencing processed with HiSeq (RNA-Seq) vs. 16S metagenomics processed with either 454-pyrosequencing (16S-454) or MiSeq (16S-MiSeq). (TIF) [file pntd.0003929.s001.tif]

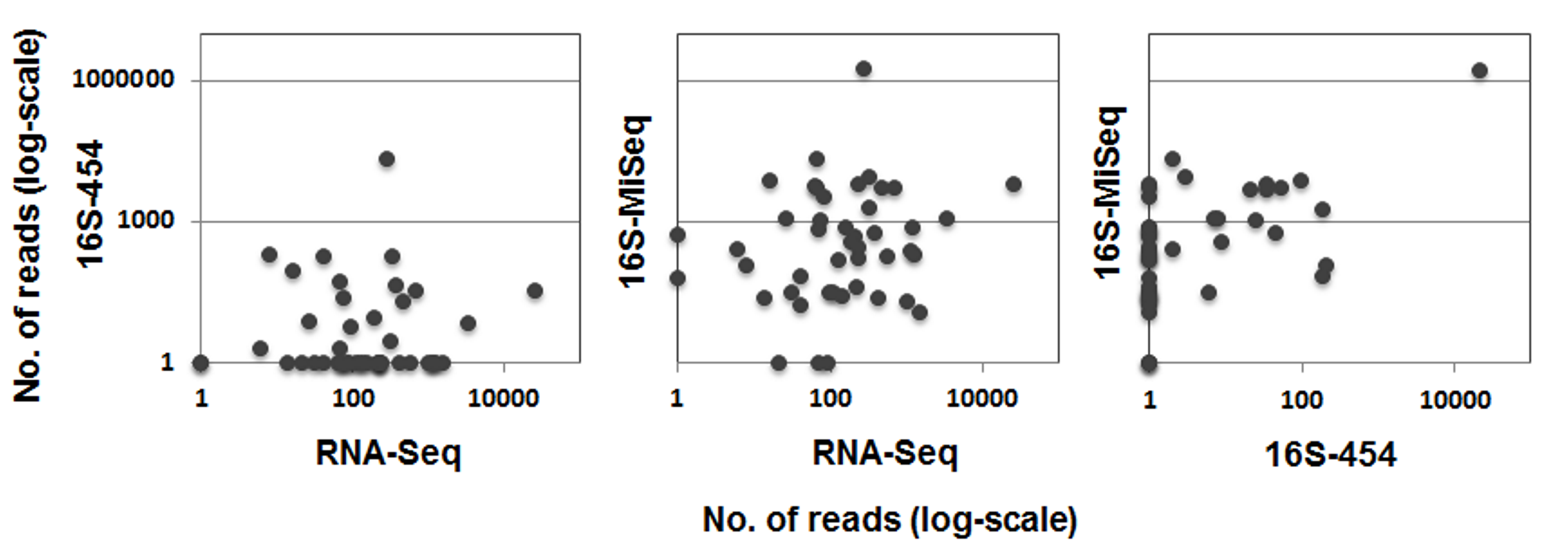

Supplement: S2 Fig — RNA sequencing processed with HiSeq (RNA-Seq) vs. 16S metagenomics processed with either 454-pyrosequencing (16S-454) or MiSeq (16S-MiSeq). Correlation coefficients (R2) and statistical significance (P) are 0.019 (P = 0.688), 0.015 (P = 0.206) and 0.293 (P<0.001), respectively. (TIF) [file pntd.0003929.s002.tif]

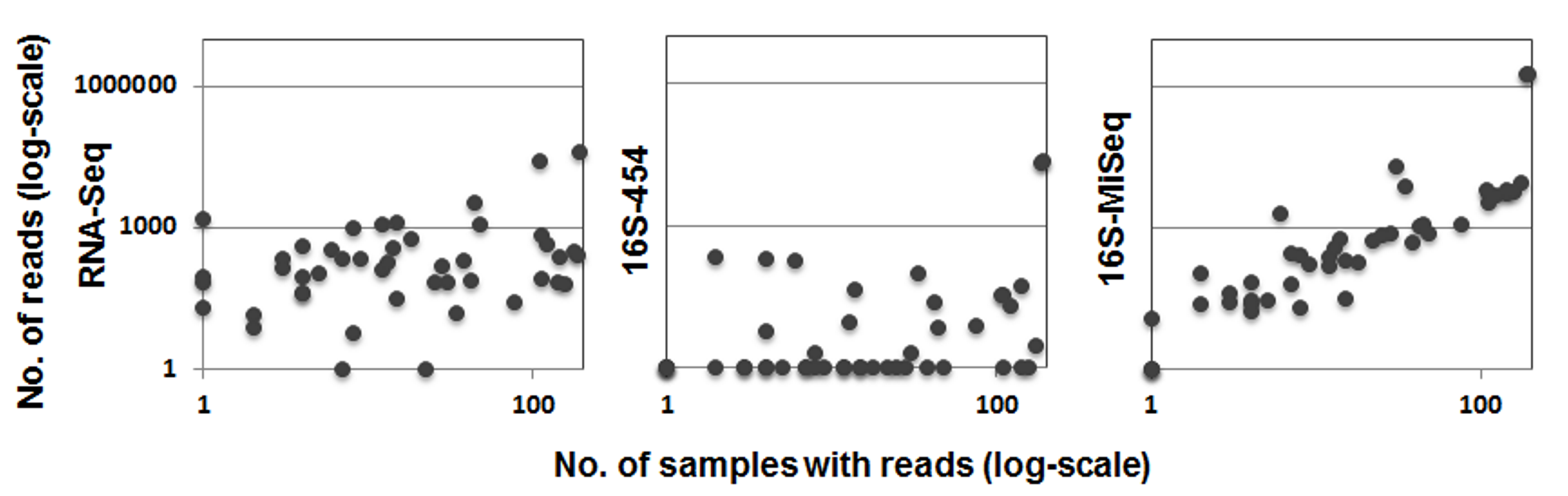

Supplement: S3 Fig — (TIF) [file pntd.0003929.s003.tif]

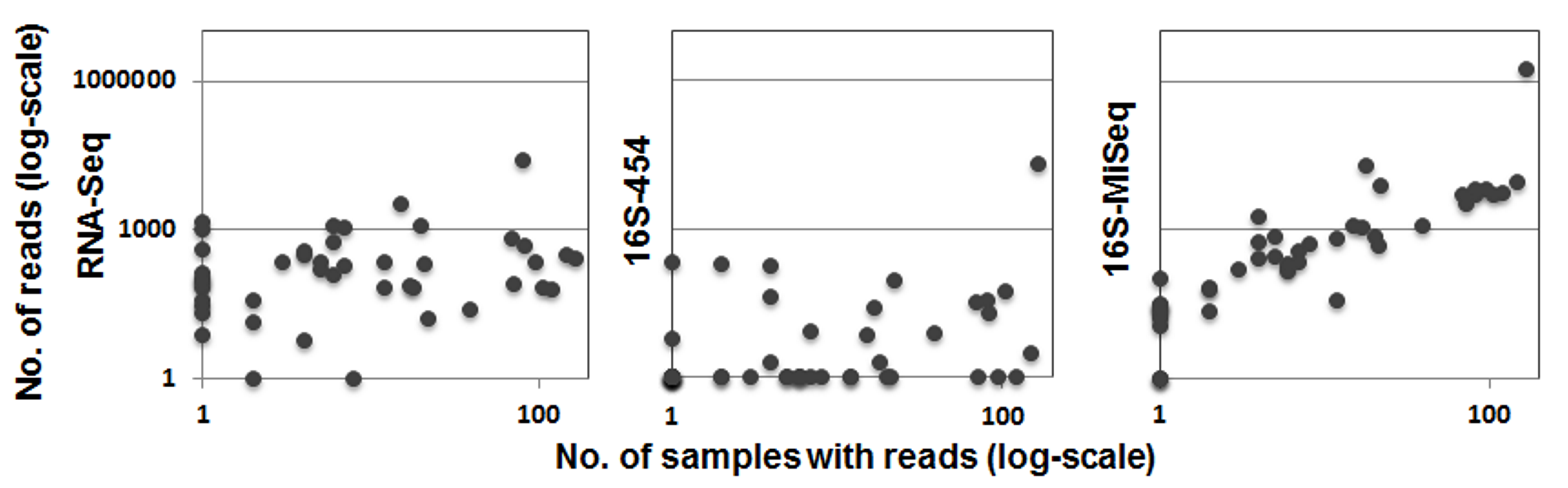

Supplement: S4 Fig — (TIF) [file pntd.0003929.s004.tif]

**% of rodents**

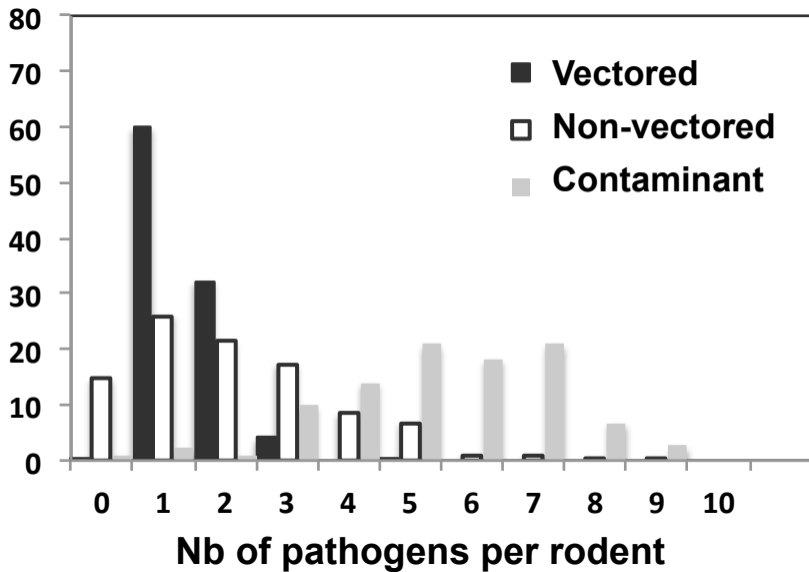

Supplement: S5 Fig — Contaminants of laboratory reagents are also shown. The results shown are from the MiSeq data. Prevalence is estimated using the number of rodent samples with at least one read. (PDF) [file pntd.0003929.s005.pdf]

% of rodents

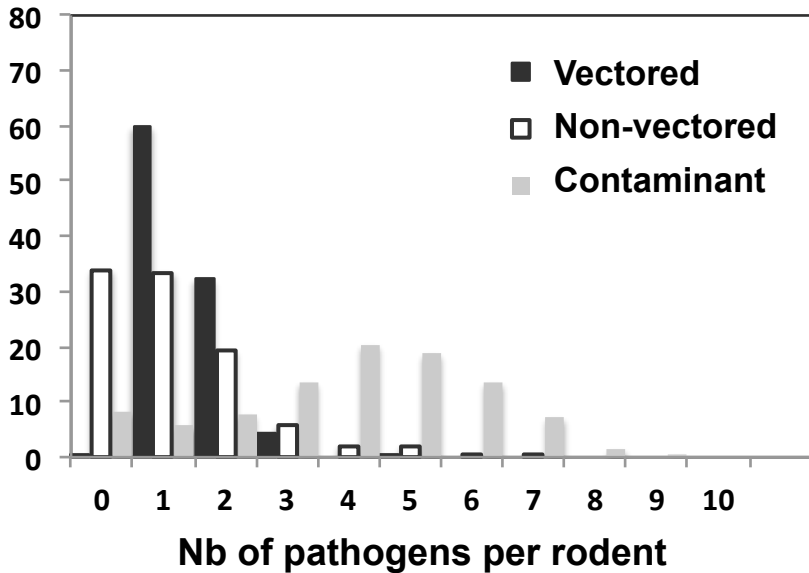

Supplement: S6 Fig — Contaminants of laboratory reagents are also shown. The results shown are from the MiSeq data. Prevalence is estimated using the number of rodent samples with at least ten reads. (PDF) [file pntd.0003929.s006.pdf]
